# Quantifying Data Quality for Clinical Trials Using Electronic Data Capture

**DOI:** 10.1371/journal.pone.0003049

**Published:** 2008-08-25

**Authors:** Meredith L. Nahm, Carl F. Pieper, Maureen M. Cunningham

**Affiliations:** 1 Duke Translational Medicine Institute, Durham, North Carolina, United States of America; 2 Department of Biostatistics and Bioinformatics, Duke University Medical Center, Durham, North Carolina, United States of America; 3 Duke Clinical Research Institute, Durham, North Carolina, United States of America; Johns Hopkins Bloomberg School of Public Health, United States of America

## Abstract

**Background:**

Historically, only partial assessments of data quality have been performed in clinical trials, for which the most common method of measuring database error rates has been to compare the case report form (CRF) to database entries and count discrepancies. Importantly, errors arising from medical record abstraction and transcription are rarely evaluated as part of such quality assessments. Electronic Data Capture (EDC) technology has had a further impact, as paper CRFs typically leveraged for quality measurement are not used in EDC processes.

**Methods and Principal Findings:**

The National Institute on Drug Abuse Treatment Clinical Trials Network has developed, implemented, and evaluated methodology for holistically assessing data quality on EDC trials. We characterize the average source-to-database error rate (14.3 errors per 10,000 fields) for the first year of use of the new evaluation method. This error rate was significantly lower than the average of published error rates for source-to-database audits, and was similar to CRF-to-database error rates reported in the published literature. We attribute this largely to an absence of medical record abstraction on the trials we examined, and to an outpatient setting characterized by less acute patient conditions.

**Conclusions:**

Historically, medical record abstraction is the most significant source of error by an order of magnitude, and should be measured and managed during the course of clinical trials. Source-to-database error rates are highly dependent on the amount of structured data collection in the clinical setting and on the complexity of the medical record, dependencies that should be considered when developing data quality benchmarks.

## Introduction

Research sponsors and clinical research organizations (CROs) are transitioning from paper-based data collection to electronic data capture (EDC) systems. If novel technologies are to be successfully integrated into clinical trials, their effects on data quality must be fully understood. Relatively few new data collection systems or methodologies, with the exception of electronic patient reported outcomes (ePRO) [1]–[12], are well-characterized with respect to data quality.

Data quality for paper-based clinical trials is traditionally assessed through audits that compare database listings against data recorded on paper case report forms (CRFs), thereby providing an estimate of the database error rate [13], [14]. Audits may also indicate location and distribution of errors, which are usually categorized in a manner meaningful to the study (e.g., critical versus noncritical) or the organization (e.g., systematic versus random errors, or according to root causes) [13]. In addition to providing objective information about processes, audits can prevent future errors by identifying problematic work patterns or behaviors.

### Clinical trial data audits

There are numerous examples, both published [15]–[33] and unpublished, of database audits that compare database listings to CRFs. The average error rate in the published literature for CRF-to-database audits is 14 errors per 10,000 fields. Such audits do not assess the percentage of correct data; rather, they identify additional errors introduced during data processing [14]. Other errors, including measurement error, recording error, or transcription mistakes that occur when transferring data from source documents to CRFs [34] lie outside the scope of traditional CRF-to-database audits. Thus, the commonly reported “database error rate” is merely an estimate of errors introduced during data entry and cleaning; at best equal to, but likely less than, the total “true” error rate. Determining actual data quality requires an assessment of all possible sources of error, including data measurement, recording, abstraction, transcription, entry, coding, or cleaning [13], [35].

In compliance with Good Clinical Practices (GCP), trial sponsors typically perform source document verification (SDV) of recorded data [36]. SDV compares original data, such as the medical record, with the study CRF. Although the SDV process is not usually quantified during trial operations, our literature review identified 42 articles that provided source-to-database error rates, primarily from registries [37]–[78]; the average error rate across these publications was 976 errors per 10,000 fields. In contrast, the average error rate for published CRF-to-database comparison audits was 14 errors per 10,000 fields [15]–[33].

With EDC, there is no paper CRF to compare to the source, leading to differences in data collection processes and resulting data quality [79]. Although EDC proponents frequently claim that clinical trial data quality improves with use of such systems, studies supporting this contention have yet to appear in the peer-reviewed literature, and it is not clear whether traditional methods of ascertaining data quality suffice for EDC trials.

### Exploring the effects of EDC on data quality

The comparison of published source-to-database and CRF-to-database error rates suggests that most errors occur when data are transferred from source to CRF during medical record abstraction or transcription. Web-based EDC can only affect the latter, through structured data collection, valid value lists, and on-screen checks for values that are missing, out of range, or inconsistent. Possible detrimental effects of EDC have not been investigated.

We sought to explore the effects, if any, of EDC on data quality. We hypothesized that for EDC to substantially improve quality, it would have to facilitate improvements to the process of medical record abstraction. Unfortunately, abstraction error rates are not usually quantified in clinical trials. Manual SDV can detect abstraction errors, but is labor intensive and highly sensitive to the vagaries of locating the pertinent text or value in medical records, leading to variability and measurement error. Additionally, ePRO systems, in which data are directly entered by the research subject, may be difficult or even impossible to assess for data quality because the information may not be validly and reliably retrieved; however, such issues lie outside the scope of our study.

### Purpose

The National Institute on Drug Abuse (NIDA) Clinical Trials Network (CTN) has instituted a process for quantifying data quality on EDC trials. In 2005, the NIDA CTN implemented the InForm (Phase Forward, Inc.) Web-based EDC system at the data and statistical center (DSC) housed at the Duke Clinical Research Institute. The system facilitates extensive error checking for missing, out-of-range, and logically inconsistent values across the CRF in real time so that many potential errors are caught prior to final data submission.

NIDA CTN trials use structured paper data collection forms as the source (patient questionnaire data for CTN trials are captured via ePRO and are not included in our analysis). The data auditing method for EDC trials provides an objective assessment of quality at each site, including sample size calculations for audits, assessment of data quality by site and by trial, corrective action processes, and reports to communicate and monitor audit results. We present findings from our initial evaluation of the NIDA CTN data quality assessment program.

## Methods

An audit plan was applied to trials conducted at the network's DSC that opened to enrollment after April 2005 and used Web-based EDC, excluding trials that were migrated to the center. Two audits, in which source data were compared to database listings for a prespecified sample of study patients, were conducted at each research site. The first source-to-database audit at each site occurred at a point when 20%–30% of the expected subjects were enrolled. The second audit was performed at 70%–80% of expected enrollment. Our audit plan incorporated both the statistically calculated sample sizes used in industry CRF-to-database audits, and the National Cancer Institute's method of auditing cases source-to-database at each site.

Researchers have choices of powering the audit based by 1) the width of the confidence interval (CI), 2) standard error, or 3) a formal hypothesis test. We considered CI and hypothesis testing methods of sample size calculation. The first CI-based method is for comparison of an error rate to a standard. Here, a known or assumed limit, or a specified acceptance criterion (Formula 1) is compared to an observed value. The intent is to ensure that the observed value is less than some criterion; hence, a one-tailed interval. The second CI-based method (Formula 2) is the CI for the difference between two sites or times; i.e., based on the standard error of the difference. The hypothesis-based method (Formula 3) is a comparison of two error rates drawn from different samples to assess differences between sites or times (e.g., error rates between two sites or between two different time points within a site).

**Figure 1:**
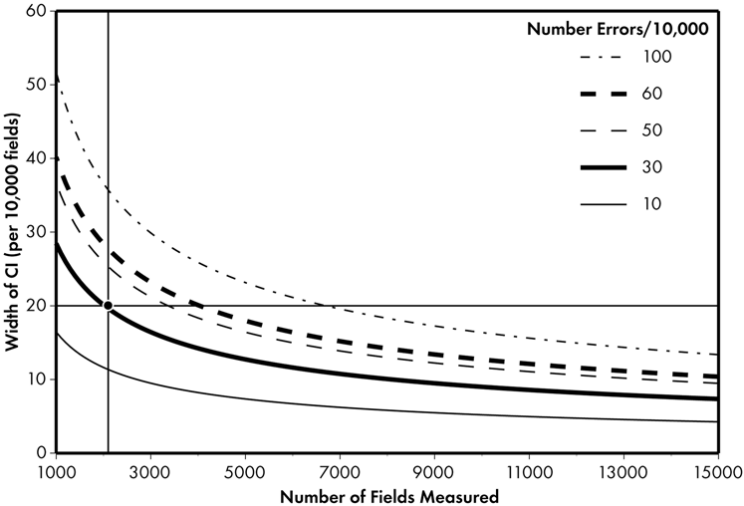
Sample size curves: 95% confidence intervals (Formula 1), one-tailed. Intersection of vertical and horizontal lines shows sample size needed to achieve a one-sided CI given an acceptance criterion of 50 errors per 10,000 data fields, an underlying expected error rate of 30 errors per 10,000 fields, and a desired CI width of 20 errors per 10,000 fields (Fleiss J, Levin BL, Paik M. Statistical Methods for Rates and Proportions. 3^rd^ ed. New York, NY: Wiley; 2003).

In the CI-based method, a one-sided CI might be used to assess the probability that the rate is lower than some prescribed level. Formula 2 would be employed to assess if a rate differed between sites or times. Assuming a 95% CI, where np_i_>5, the CI can be calculated from the following equations [80]:
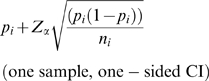
(1)

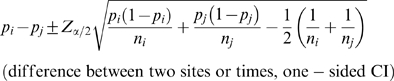
(2)where Z_α_ is 1.645 (the one-sided alpha level associated with 95% of the normal Z), and p_i_ is the observed error rate in site, i, of sample size n_i_. Further, in Formula 2, p_j_ is the error at site or time j of size n_j_ to be compared to some other error rate p_j_ with sample size n_j_, and Z_α/2_ is 1.96 (the two-sided alpha level associated with 95% of the normal Z). As written, the formulas show a CI for a given p_i_ (and p_j_) and sample size. The required sample size can then be algebraically derived. Sample size curves for a variety of desired CI widths and expected error rates are shown in Figure 1. The sample size based on a one-sided CI for an acceptance criterion of 50 errors per 10,000 fields, underlying expected error rate of 30 errors per 10,000 fields, and a desired CI width of 20 errors per 10,000 fields, is 2100 fields. Curves for difference-based CIs (Formula 2) can be similarly derived.

**Figure 2:**
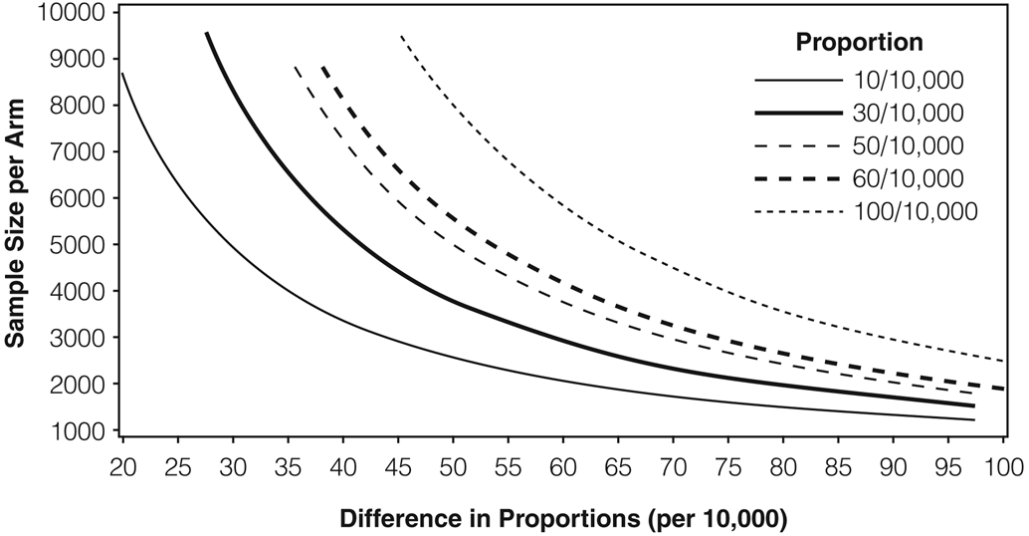
Sample size curves: Hypothesis testing method (Formula 3) at 80% power and α of 0.05 (two-tailed), showing sample sizes needed to distinguish among groups for given baseline error rates and assumed differences (Fleiss J, Levin BL, Paik M. Statistical Methods for Rates and Proportions. 3^rd^ ed. New York, NY: Wiley; 2003).

A formal hypothesis test could be conducted; e.g., to test if there are differences between sites or times. The test of a difference in error rates between two sites or times requires a slightly different formula: p_i_ and p_j_ are averaged under the null hypothesis to give Formula 3:
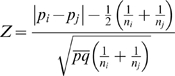
(3)where *p̅* is the average of p_i_ and 
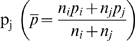
 and *q̅* = 1−*p̅*. The required sample size can then be algebraically derived at a given error rate and assumed difference (p_i_ and p_i_−p_j_). This test, however, does not adjust for power, nor for multiple comparisons. As shown in Figure 2, for a set of baseline error rates and assumed differences, the sample size required to distinguish groups quickly becomes large at 80% power. For example, if the error rate is 30/10,000 in one site, and the error rate in the second site is triple (difference = 60), then at 80% power (and not adjusting the overall type I error rate for multiple comparisons), we would require 2900 fields per group. Where n_i_p_i_<5, the normal approximation breaks down. Since many audits have found n_i_p_i_<5, and since as n_i_p_i_ increases, exact methods approach those using the normal approximation, we employed the Clopper-Pearson exact method [81] to calculate the CIs presented in the Results section.

We sought to detect differences between sites. Given CTN site norms (i.e., outpatient setting, patients whose conditions are chronic rather than acute, and significant use of structured worksheets for source data collection), we assumed a rate of 50 errors per 10,000 fields, and wished to obtain a 20 error per 10,000 field CI, yielding a sample size of 3400 fields per site.

A sample of at least 3500 data fields, providing 100 fields overage, was obtained by selecting random forms (a CRF page or subset of pages from a patient visit) from the list of patient forms. An additional 3500 fields were audited when approximately 70%–80% of expected enrollment was achieved, providing a statistically representative sample at each site across two time points. Any discrepancy between source and database not explained by study documentation was counted as an error. The error rate denominator was the number of fields actually audited, excluding those defined as system-calculated or propagated fields.

## Results

**Figure 3:**
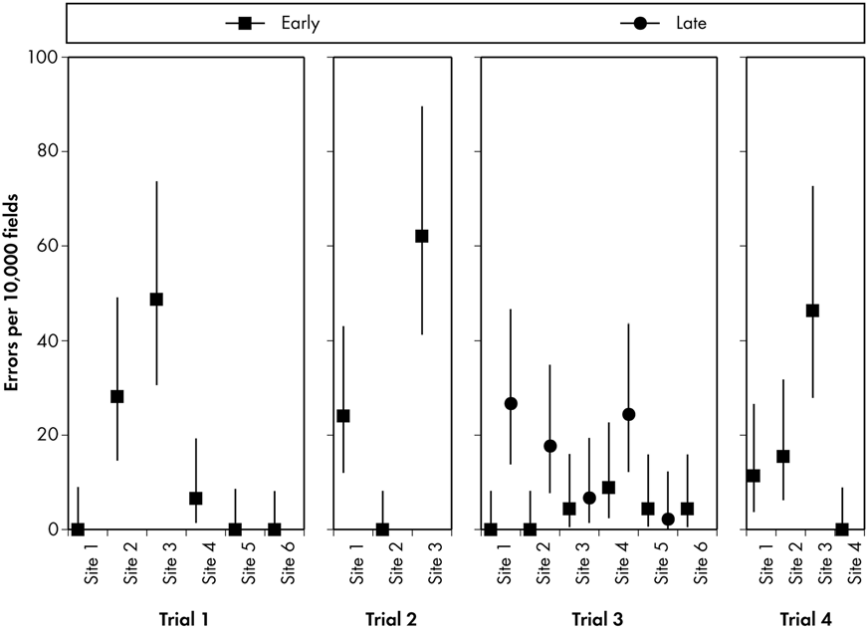
Source-to-Database Audit Error Rates for CTN EDC Trials 1–4. The first source-to-database audit (“early”) was performed when 20%–30% of expected subject enrollment was reached; the second database audit (“late”) was performed when 70%–80% of expected enrollment was reached.

For our initial assessment, we completed source-to-database audits of 24 sites participating in 4 EDC trials conducted through the CTN (Figure 3). Preliminary findings show an average error rate across all 4 trials of 14.3 errors per 10,000 fields, with a 95% CI (averaged across audit CIs) of 12–39 per 10,000 fields, a low rate compared with those reported for source-to-database audits, and comparable to the average of reported CRF-to-database error rates. Fourteen percent of errors were in fields critical to the analysis (major independent or dependent variables or covariates).

**Figure 4:**
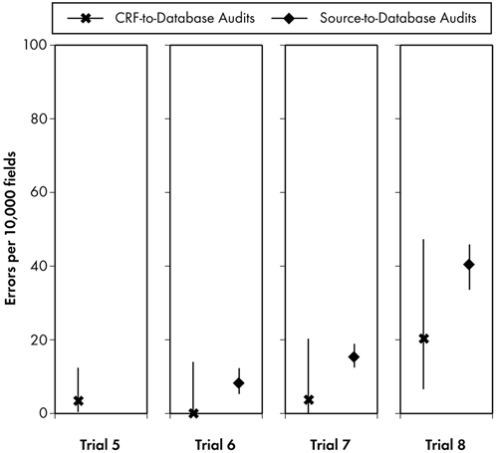
Source-to-database and CRF-to-database audits for Comparator Trials 5–8. These audits were undertaken to provide a “control” for comparison with Trials 1–4 (results displayed in Figure 3).

Because these results, which were considerably lower than published error rates for source-to-database audits, seemed counterintuitive, we compared them to audit results from four earlier paper-based trials managed at the DSC (Figure 4). Three trials—5, 6, and 7—used paper CRFs sent to the DSC for double data entry and cleaning. Trials 5, 6, and 7 had error rates of 3.4, 0, and 3.7 errors per 10,000 fields, respectively, as determined by CRF-to-database audits. Trial 5 used only CRF-to-database auditing at the DSC. Trials 6 and 7 were migrated to the DSC and audited both source-to-database (as part of ongoing quality control) and CRF-to-database to measure processing fidelity for migrated data. The source-to-database error rates for Trials 6 and 7 were 8.3 (5, 13) and 15.4 (13, 19) errors per 10,000 fields, respectively (Figure 4).

Trial 8 was also migrated to the DSC but employed a form of Web-based EDC in which sites completed paper CRFs and transcribed data into the EDC system. Legacy data were single-entered at the DSC from printed data listings and subjected to “CRF” (data listing)-to-database audits to assess fidelity of data processing. The error rate for this migrated data was 20.3 (7, 50) errors per 10,000 fields. The source-to-database error rate for Trial 8 was 40.5 (36, 46) errors per 10,000 fields (Figure 4). We attribute the difference between Trials 5, 6, and 7 as compared to Trial 8 to the data processing method used for the latter trial. Comparison of Figures 3 and 4 shows that the source-to-database and CRF-to-database audit results are comparable.

During this period, the DSC also performed a source-to-database audit for a trial in a different therapeutic area (epilepsy). This study, characterized by medically complex patients, an inpatient phase, and a more complex medical record, proved a useful comparator to CTN protocol trials. Data were abstracted directly from medical records and entered. A total of 3250 data fields from five subjects were audited. We identified 139 errors, yielding an estimated error rate of 428 errors per 10,000 fields, comparable to the published literature for source-to-database audits.

## Discussion

The CTN source-to-database error rates were unexpectedly low, especially when compared to the average of 976 errors per 10,000 fields derived from published reports for source-to-database audits, and the rate of 428 errors per 10,000 fields from a recent source-to-database audit conducted at our center. The CTN source-to-database results more closely resembled CRF-to-database error rates reported in the literature and touted by industry.

One reason for these unexpected results may be the processes used to document treatment at NIDA CTN sites. CTN sites are community treatment programs for substance abuse and addiction treatment. In this setting, patient charts, largely consisting of clinic notes, tend to be brief, and confidentiality policies restrict access to research records. The CTN sites therefore separate subjects' research and clinic records, with study visit documentation residing in the research record. Because more data typically are collected during clinical research than in standard practice, and because some programs do not clinically document treatment, worksheets provided to sites for capturing trial data often comprise the source documents. Data from these worksheets are single-entered by site staff into an EDC system with extensive on-screen checking.

In this context, our results are consistent with previous reports, with CRF-to-database error rates being lowest, followed by EDC data entered from worksheets, and finally the source-to-database error rate from therapeutic areas characterized by more acute patient conditions being highest. However, we emphasize that our findings are derived from a specialized and somewhat atypical clinical research environment; given the wide variability in the design and conduct of clinical trials, our results may not be generalizable to other research venues and should be viewed as hypothesis-generating only.

We examined variations in local monitoring by regional and community treatment centers, some of which undertake additional quality assurance. Two trials employed a central quality assurance (QA) monitor who performed SDV at all participating sites; one trial required sites to have local QA auditors to perform SDV; another neither performed central monitoring nor required sites to do so. We expected pseudo-independent central monitoring to produce higher-quality data than decentralized monitoring, and a decentralized monitoring regime to produce higher-quality data than no monitoring. However, we observed no correlation between database error rates and differences in additional local auditing or monitoring. In the clinical trials arena, source document verification (manual comparison of the medical record to the CRF or database) although unproven, is generally thought to decrease data errors. Effective SDV would be a confounding factor impacting data error rates, and should be taken into account when interpreting results.

Anticipation of an audit may be an important quality assurance mechanism, providing sites an additional incentive to maintain data quality. Based on observed error rates, one audit visit per site may be sufficient, as statistical power remains suffices for determining the data quality of the trial as a whole as well as at each site.

Given these findings, the NIDA CTN changed its auditing plan and decreased the frequency of audits, resulting in reduced travel expenses incurred by auditors. Under the revised plan, an initial source-to-database audit would be performed for each site upon reaching 20%–30% of expected enrollment. Sites with an error rate over 50 errors per 10,000 fields would require a second audit. Most sites with error rates below this benchmark that also addressed data queries and protocol violations in a timely manner would not receive additional audits, although one site would be chosen at random for a second audit. The revised plan results in fewer logistical and financial burdens for sites, should continue to provide comprehensive data quality monitoring, and could potentially prove more cost-effective, although without accurate comparators, this assertion remains speculative. An alternative approach, in which sites would be given 24 hours to copy specified charts and send them to the data center, was considered but deemed more burdensome by the sites.

It is also worth noting that as electronic health records (EHRs) become increasingly ubiquitous, clinical researchers may adopt data collection strategies that directly access patient medical records, which would streamline the process of data collection and may significantly reduce errors associated with medical record abstraction. Such strategies, however, will face a number of hurdles, including electronic access to patient data by research staff, information retrieval, privacy concerns, and issues relating to data standardization.

### Acceptance criteria

NIDA CTN sites initially requested that an acceptance criterion be set in order to provide an objective performance standard; however, the authors felt that the introduction of such a criterion at the onset of the audit program was premature and not justified by an appropriate basis in evidence. Instead, we compared sites within a trial and performed an assessment, described here, early in the program to investigate the applicability of a CTN-wide acceptance criterion.

All random errors detected during source-to-database audits were reviewed with the site and subsequently corrected. If an error was deemed systematic (i.e., occurring across subjects or forms and due to a common cause), the characteristics and root cause were used to identify similar occurrences and apply corrections throughout the database. Error rates within a trial were also compared across sites to identify sites whose data quality differed substantially from others'. If the error rate of source-to-database audits for a given site was outside the bounds of the 95% CI calculated over all audits on the trial, that site's data quality was deemed to differ sufficiently to warrant further investigation and intervention. In such cases, site data were further examined to elucidate the source of the errors, and corrective action was taken to bring data quality within range of other sites. We also compared error rates by trial to explore differences in data quality across trials.

Setting an acceptance criterion is unnecessary from a statistical point of view, given that 95% CIs could be used. Two possibilities then arise: 1) if any site's error rate is above the upper bound of the overall CI (aggregated across all sites, calculated from the total number of audited fields across all sites and the total number of errors across all sites for a trial), the error rate may be considered excessive, or 2) if any site's CI exceeds the upper bound of the overall CI, the site's error rate would be considered excessive. However, such rules may fail to produce operationally meaningful results; e.g., differences between sites might be so small as to have no effect on conclusions drawn from the trial, if all sites had relatively low error rates and consistently narrow CIs. Such methods might also promote a competitive or even punitive environment.

A useful acceptance criterion, then, would distinguish operationally meaningful differences. Now that we understand the process capability of the CTN, naming an acceptance criterion would also: 1) provide sites with objective performance benchmarks, allowing sites to alter internal quality systems accordingly; 2) allow statisticians to assess its appropriateness for that particular trial; and 3) provide a common language for trial-specific needs to be communicated to sites.

The consistency of data from three of the 4 trials implies that a “network-wide” acceptance criterion could be set. CTN sites were able to meet our relatively arbitrary limit of 50 errors or fewer per 10,000 fields; there was no indication that this limit was excessive. Even though we measured source-to-database processes, it is reassuring that this limit is within industry expectations for CRF-to-database processes. A recent data quality survey conducted by the Society for Clinical Data Management reported the most popular overall database error rate acceptance criteria to be 50 errors per 10,000 fields and 10 errors per 10,000 fields. The most popular acceptance criteria for critical variables were 10 errors per 10,000 fields and zero errors per 10,000 fields [34]. The question of “how many errors is too many?” is difficult to answer because it depends on many factors, including what variables are in error, the robustness of the analysis, and the concern that a single data error may cast doubt on the validity of the rest of the data [82]. Thus, arbitrary (and low) acceptance criteria tend to be employed.

We opted not to re-audit sites whose upper CIs exceeded our established limit, thereby accepting the level of risk implicit within the CI. However, in a situation in which many CTN sites were participating in multiple trials, if a particular site consistently appeared close to the limit of the acceptance criterion, those findings could be addressed as a trend. During the initial program, one trial had a significantly higher error rate than the others. A single site was identified as the cause of the high error rate; that site also had a significantly higher rate of protocol violations, and suffered most frequently from computer-related problems.

Operationally, the initial 3500-field sample size allowed for a half day on site, but the requirement to complete the audit at 20%–30% of enrollment at each site did not permit trips to be combined. The benchmark of 20% enrollment was selected to ensure that sufficient data were available for source-to-database audits, but that the amount would be too large for sites to “scrub” the first few cases. Conducting audits sufficiently early for sites to benefit from using results to prevent future problems and to allow sufficient time for remediation were also significant considerations, as such instant feedback appeared to promote more effective site management.

### Limitations

Our results are limited to a single therapeutic area and are drawn from a setting that may not be generalizable to other arenas. Our results, however, may not be extrapolable to inexperienced research sites, or to therapeutic areas that require significant amounts of medical record abstraction, or to industry trials that lack CTN research infrastructure. Further, these results are based on our experience with a single commercial EDC system; use of a different system, or variations in implementation of the same system, might have a significant impact on data quality, and methods for calculating error rates vary widely across the industry [13], [34].

Important questions remain to be answered, however; for example, the impact of data cleaning and auditing on trial results remains unclear. In addition, a model does not yet exist for error distributions in clinical trial data. In the absence of such a model, event independence is assumed (e.g., in our sample size calculations).

### Conclusions

Our evaluation provides additional evidence that medical record abstraction and transcription are the steps most likely to introduce error into data collection and management processes, and that source-to-database error rates may vary depending on therapeutic area and according to site data practices. Data centers should be aware of these factors, and provide assistance to sites in reducing variability in the abstraction process. We also found that the capacity to compare data at the level of individual sites facilitated evaluation and allowed us to demonstrate the degree of consistency among sites. Finally, we observed that higher error rates may correlate with other operational problems. We believe that objectively quantifying data quality will provide a more comprehensive picture of a site's performance.
